# Spatial heterogeneity in area-level environmental context of hospital-based congenital structural anomaly burden in Southwest China: a retrospective study from a provincial pediatric referral center

**DOI:** 10.1007/s12519-026-01059-w

**Published:** 2026-09-03

**Authors:** Cheng-Hao Zhanghuang, Yong-Yu Ma, Chun-Li Zheng, Xi Hu, Ming-Xiang Zhang, Yun-Peng Gao, Jun-Ru Chen, Shi-Wu Yang, Hong Zhang, Ru-Tao Dai, Xi-Chen Zhang, Jin Shen, Bing Yan, Jun Wu

**Affiliations:** 1 Department of Cardiothoracic Surgery, Children’s Hospital affiliated to Kunming Medical University (Kunming Children’s Hospital), Kunming, 650103 China; 2 Yunnan Province Clinical Research Center for Children’s Health and Disease, Yunnan Key Laboratory of Children’s Major Disease Research, Kunming, 650103 China; 3 Department of Urology, Children’s Hospital affiliated to Kunming Medical University (Kunming Children’s Hospital), Kunming, 650103 China; 4 Geography Department, Yunnan Normal University, Kunming, 650500 China; 5 Department of Emergency Trauma Surgery, Children’s Hospital affiliated to Kunming Medical University (Kunming Children’s Hospital), Kunming, 650103 China; 6 Department of Medical Record Management, Children’s Hospital affiliated to Kunming Medical University (Kunming Children’s Hospital), Kunming, 650103 China

**Keywords:** Congenital abnormalities, Environmental context, Epidemiology, Pediatrics, Spatial analysis

## Abstract

**Background:**

Congenital structural anomalies constitute a major source of pediatric morbidity and surgical burden. However, evidence regarding the spatial epidemiology of congenital structural anomalies and their area-level environmental context remains limited, particularly in geographically and socioeconomically heterogeneous regions. This study aimed to investigate the spatial heterogeneity of area-level environmental contextual associations with hospital-based congenital anomaly burden.

**Methods:**

We conducted a retrospective hospital-based study including pediatric inpatients with congenital structural anomalies admitted to the largest provincial pediatric referral center in Yunnan Province between 2014 and 2024. Descriptive epidemiological analyses were performed to summarize demographic characteristics, temporal trends, and system-level distributions. High-frequency congenital anomalies were selected to construct stable county-level datasets for spatial analysis. Ordinary least squares (OLS) and geographically weighted regression (GWR) models were applied to examine spatially varying, area-level contextual associations between environmental factors and anomaly burden across five congenital anomaly systems (circulatory, digestive, urogenital, musculoskeletal, and other structural anomalies).

**Results:**

A total of 56,434 pediatric inpatients with congenital structural anomalies were identified. Among pediatric inpatients, boys accounted for the majority (67.68%), and admissions were predominantly concentrated in early childhood. Digestive (29.63%) and urogenital (23.30%) anomalies accounted for the largest proportions of pediatric inpatients. Among 41,531 pediatric inpatients included in spatial modeling, GWR consistently demonstrated better performance than global OLS models across five congenital anomaly systems. Area-level environmental associations with referral-weighted institutional burden showed substantial spatial heterogeneity. Carbon monoxide demonstrated predominantly positive associations across anomaly systems, whereas sulfur dioxide showed pronounced spatial heterogeneity. Vegetation coverage showed consistent negative associations, while population density displayed geographically varying associations.

**Conclusions:**

Hospital-based congenital structural anomaly burden showed a substantial spatial heterogeneity in its area-level environmental context within a provincial pediatric referral center dataset. Spatially explicit approaches such as GWR may help characterize referral-weighted, area-level patterns of institutional burden beyond global models. These findings are descriptive and hypothesis-generating and require validation using population-based or multi-center studies.

**Graphical abstract:**

**Figure:**
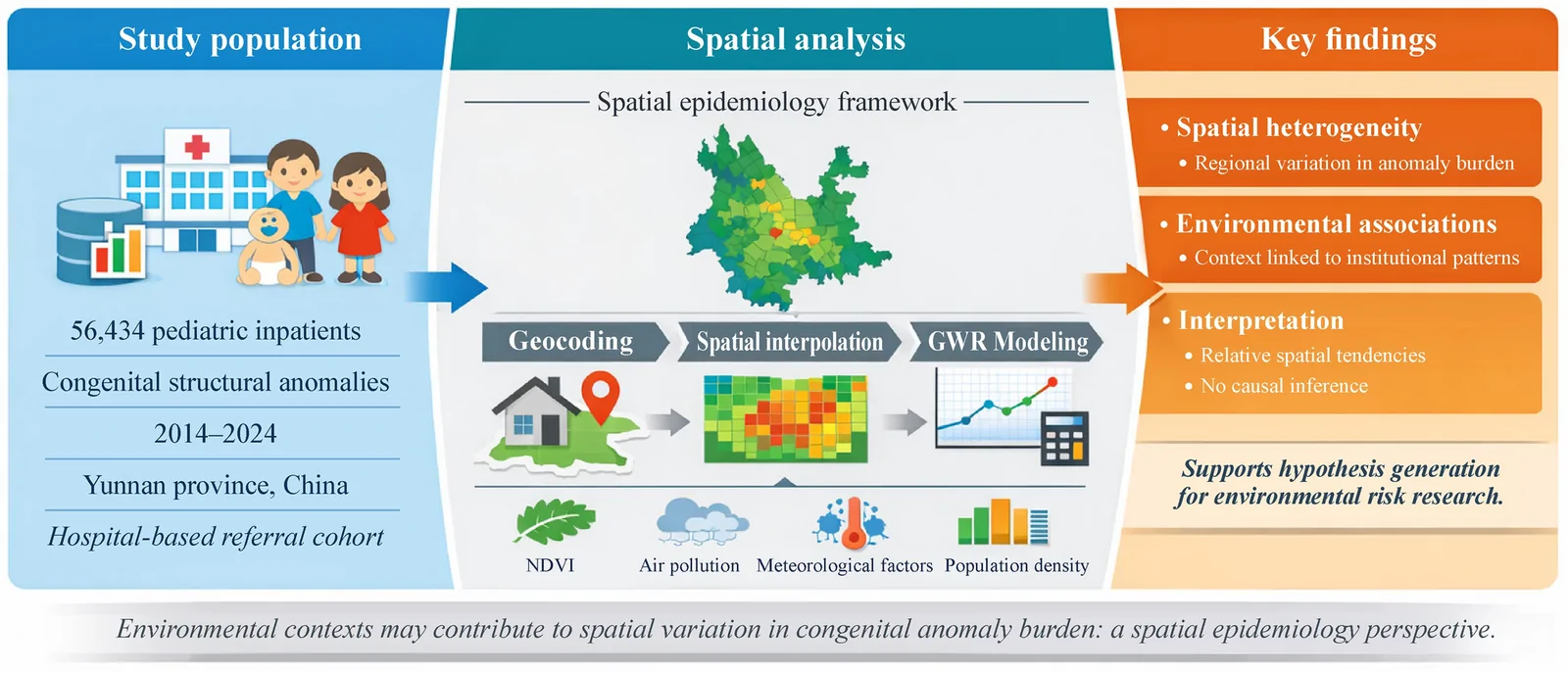

**Supplementary Information:**

The online version contains supplementary material available at https://doi.org/10.1007/s12519-026-01059-w.

## Introduction

Congenital structural anomalies are among the leading causes of infant morbidity, long-term disability, and pediatric surgical intervention worldwide [1, 2]. Although advances in prenatal screening and pediatric surgery have substantially improved early diagnosis and clinical outcomes, the etiological pathways underlying congenital anomalies remain complex and incompletely understood [3]. In addition to well-established genetic and developmental determinants, increasing attention has been directed toward the role of environmental exposures, particularly ambient air pollution and broader environmental contexts, in influencing fetal development and congenital anomaly risk [4, 5].

Most existing epidemiological evidence on environmental risk factors for congenital anomalies is derived from global or national-level analyses that implicitly assume spatially stationary associations [6]. However, environmental exposures, socioeconomic conditions, and healthcare access often vary substantially across regions, particularly within geographically heterogeneous settings. Ignoring such spatial heterogeneity may obscure localized vulnerability and limit the effectiveness of prevention strategies [7]. Spatial epidemiology focuses on the analysis of health outcomes and environmental contexts at the area level, aiming to identify geographic heterogeneity in disease burden and contextual associations. Unlike individual-level epidemiological studies, spatial approaches are not primarily designed to establish causal relationships at the individual level, but rather to characterize regional patterns, contextual vulnerability, and spatial non-stationarity. In this framework, area-level environmental indicators serve as proxies for long-term background exposure and contextual conditions, providing insights into geographically structured health disparities that may help generate hypotheses for future surveillance-oriented or population-based studies [8, 9].

China exhibits pronounced regional diversity in geography, industrial structure, and environmental exposure profiles [10]. Yunnan Province, located in Southwest China, is characterized by complex terrain, uneven economic development, and marked urban–rural contrasts, making it a compelling setting for investigating spatial variability in environmental health associations [11, 12]. As the largest tertiary pediatric referral center in the province, our institution receives children from both urban and remote rural areas, providing an opportunity to examine hospital-based clinical patterns and referral-related healthcare demand across the region. In this context, the present study combined descriptive epidemiology with geographically weighted regression (GWR) to examine county-level patterns of hospital-based congenital structural anomaly burden across five major organ systems in Yunnan Province. Specifically, we assessed whether associations between long-term county-level environmental indicators and referral-weighted institutional burden varied geographically across the province, rather than assuming a single uniform association. This approach was intended to describe spatial heterogeneity and generate hypotheses for future population-based or multi-center studies, rather than to infer individual-level causality.

## Methods

### Study design and population

This retrospective, hospital-based study included pediatric inpatients with congenital structural anomalies admitted to the largest provincial pediatric referral center in Yunnan Province between January 2014 and December 2024. The study period refers to years where patients were hospitalized rather than their birth years. As a single-center inpatient dataset, the findings reflect healthcare-seeking and referral patterns to our institution and should not be interpreted as province-wide prevalence estimates. All diagnoses were coded according to the International Classification of Diseases, 10th Revision (ICD-10). Congenital anomalies were identified based on ICD-10 discharge diagnoses recorded in the hospital information system. Accordingly, the present analysis focuses on institutional disease burden and spatial patterns of healthcare utilization, rather than estimating population-level incidence or prevalence. Detailed genetic diagnostic information was not consistently available for all patients and, therefore, genetic etiologies could not be reliably excluded.

Because this study was based on a single provincial pediatric referral center, the dependent variables used in the spatial analysis represent institution-based disease burden and referral-weighted healthcare utilization at the county level, rather than population-based incidence or risk. These measures are inherently influenced by healthcare-seeking behavior, referral pathways, accessibility, and institutional admission practices. Accordingly, all spatial associations reported in this study should be interpreted as contextual, area-level patterns of institutional burden rather than individual-level or population-level disease risk. The hospital information system used in this study is subject to routine quality control procedures, including the verification of diagnostic coding and audit of electronic medical records. This supports the completeness and reliability of our clinical dataset.

### Descriptive epidemiology

Descriptive analyses were first conducted to characterize the demographic structure and system distribution of congenital anomalies in the entire inpatient cohort. Sex was summarized as counts and proportions. Age was categorized into six groups (≤ 28 days, 29 days to < 1 year, 1 to < 3 years, 3 to < 6 years, 6 to < 12 years, and ≥ 12 years). Annual admission trends from 2014 to 2024 were examined to assess temporal changes in hospitalization burden. Ethnicity was summarized according to routinely recorded ethnic categories. Congenital anomalies were further classified into five major systems based on ICD-10 chapters: circulatory, digestive, urogenital, musculoskeletal, and other structural anomalies. Neurological anomalies were not included in the present analysis because the number of patients managed at our institution was relatively small compared with other congenital anomaly categories. Moreover, further subdivision into specific neurological diagnoses would result in limited sample sizes and unstable spatial estimates. Therefore, to ensure model reliability and interpretability, neurological anomalies were not included in the system-level spatial analysis. Because the unit of analysis was the individual patient rather than each anomaly, patients with multiple congenital structural anomalies were assigned to a single system according to the principal discharge diagnosis and were not counted repeatedly across systems. The principal diagnosis reflected the primary reason for hospitalization and, as a standardized mandatory field, provided the most complete and reproducible classification for this retrospective dataset. This approach also reduced information bias because some critically ill patients deteriorated or died before complete multisystem evaluation. Accordingly, this analysis describes the system distribution of hospitalized patients rather than the burden of all anomaly lesions.

### Spatial analysis dataset for geographically weighted regression

Because GWR requires adequate spatially distributed data, a frequency-based disease filtering strategy was applied to construct a robust spatial analysis cohort. From the initial 56,434 pediatric inpatients, only the most frequent diseases within each system were retained (top three diseases in circulatory, urogenital, and other structural systems; top four diseases in digestive and musculoskeletal systems). The retained diseases were as follows: circulatory system (atrial septal defect, ventricular septal defect, patent ductus arteriosus); urogenital system (cryptorchidism, other congenital penile anomalies, hypospadias); digestive system (ankyloglossia, anorectal malformations, Hirschsprung disease, other tongue anomalies); musculoskeletal system (polydactyly, developmental dysplasia of the hip, congenital talipes equinovarus, pectus excavatum); and other structural anomalies (cleft lip/palate, preauricular sinus/cyst, branchial cleft anomalies).

This frequency-based filtering yielded a final GWR dataset of 41,531 patients and was used to reduce county-level zero inflation and improve the numerical stability and interpretability of the spatial regression models, rather than to prioritize diseases according to clinical severity or outcome relevance.

### Spatial units and dependent variables

All spatial analyses were conducted at the county level. The administrative boundaries of the study area were derived from the standard map downloaded from the Standard Map Service website of the Ministry of Natural Resources of the People's Republic of China [review number: GS(2023)2767], and the boundaries of the base map were not modified. In accordance with the relevant map-publication requirements, the map figures used a linear scale bar. For each system, the dependent variable was defined as a county-linked measure of referral-weighted institutional burden based on retained high-frequency anomalies, rather than a population-based prevalence estimate.

### Environmental exposure variables

All environmental variables were calculated as long-term county-level averages spanning 2014–2023, broadly covering the temporal range of the clinical dataset. These measures were intended to reflect long-term background environmental context rather than time-specific prenatal exposure windows. County-level air pollutant exposures were derived from publicly available gridded environmental datasets and spatially matched to county-level health data. Specifically, carbon monoxide (CO), PM_2.5_, PM_10_, ozone (O_3_), and land surface temperature were obtained from the National Tibetan Plateau Data Center; sulfur dioxide (SO_2_) and nitrogen dioxide (NO_2_) were obtained from Zenodo China High series datasets; carbon dioxide (CO_2_) was obtained from the Emissions Database for Global Atmospheric Research; population density was obtained from the LandScan dataset; and elevation was obtained from OpenTopography. Normalized difference vegetation index (NDVI) was derived from the MOD13A3 dataset, formally named MODIS/Terra Vegetation Indices Monthly L3 Global 1 km SIN Grid, accessed through NASA Earthdata, and was aggregated to the county level for spatial modeling. Initially, a total of 11 environmental and contextual variables were considered, including SO_2_, CO, PM_2.5_, PM_10_, O_3_, NO_2_, NDVI, land surface temperature, elevation, CO_2_, and population density. To address multicollinearity, variance inflation factors (VIFs) were calculated, and variables with VIF > 10 were excluded from the final models. Additional details on contextual variable handling and data sources are provided in the Online Supplementary Material.

### Geographically weighted regression

Ordinary least squares (OLS) models were first fitted for each system as global benchmark models. GWR was then applied to allow regression coefficients to vary spatially across counties. System-specific GWR models were fitted to account for biological and clinical heterogeneity across anomaly systems, which may obscure system-level spatial patterns in aggregated analyses.

GWR models were specified assuming a Gaussian distribution with an adaptive kernel. Adaptive bandwidths were selected using a golden section search procedure by minimizing the corrected Akaike information criterion (AICc). Model performance was evaluated using coefficients of determination (*R*^2^), AICc, and cross-validation (CV). The primary objective of this analysis was to characterize spatial variation in associations rather than to establish causal relationships. Therefore, multivariate models were used to capture contextual environmental associations. Mapped local coefficients are presented for descriptive exploration and hypothesis generation only. Formal statistical inference at individual county locations, including multiple-comparison-adjusted significance testing, was not performed.

In the Gaussian GWR framework, the fitted values (*ŷ*) were used to characterize the relative spatial tendency of congenital anomaly burden after accounting for environmental and contextual covariates. These fitted values do not represent disease risk, incidence, or individual susceptibility. Because regression coefficients were allowed to vary spatially, the fitted values represent location-specific expected levels rather than individual risk or incidence. For interpretability, *ŷ* values were standardized and mapped to illustrate spatial patterns of relative spatial tendency of institutional burden. This should be interpreted as a comparative, model-based index rather than an absolute measure of disease risk. Further details regarding the interpretation of Gaussian GWR fitted values and the descriptive composite vulnerability index are provided in the Online Supplementary Material.

In addition, a descriptive composite vulnerability index was constructed by integrating selected environmental and population characteristics for visualization purposes only, aiming to illustrate regional differences in baseline relative spatial tendency of institutional burden rather than to generate predictive estimates. For model reporting, the number of analytic observations shown in Table 2 refers to the final preprocessed observations entered into each system-specific GWR model and should not be interpreted as the number of counties or unique patients.

## Results

### Overall descriptive epidemiology

From January 2014 to December 2024, a total of 56,434 pediatric inpatients with congenital structural anomalies were identified at our institution in Yunnan Province. These cases represent the inpatient caseload of a single provincial pediatric referral center and, therefore, reflect institutional disease burden and referral-related patterns, rather than the overall provincial incidence or prevalence of congenital anomalies.

Amongst the included patients, 38,197 (67.68%) were boys and 18,237 (32.32%) were girls, with a male-to-female ratio of approximately 2.1:1 (Table 1).

**Table 1:** Demographic characteristics of pediatric inpatients with congenital structural anomalies, 2014–2024 (*N* = 56,434)

Variables	*n*	%
Sex		
Male	38,197	67.68
Female	18,237	32.32
Age group		
≤ 28 d	3071	5.44
29 d to < 1 y	10,839	19.21
1 to < 3 y	16,305	28.89
3 to < 6 y	14,455	25.61
6 to < 12 y	10,136	17.96
≥ 12 y	1628	2.89
Major system anomalies		
Digestive	16,722	29.63
Urogenital	13,147	23.30
Musculoskeletal	9742	17.26
Circulatory	6866	12.17
Other structural	9957	17.64

**Fig. 1:**
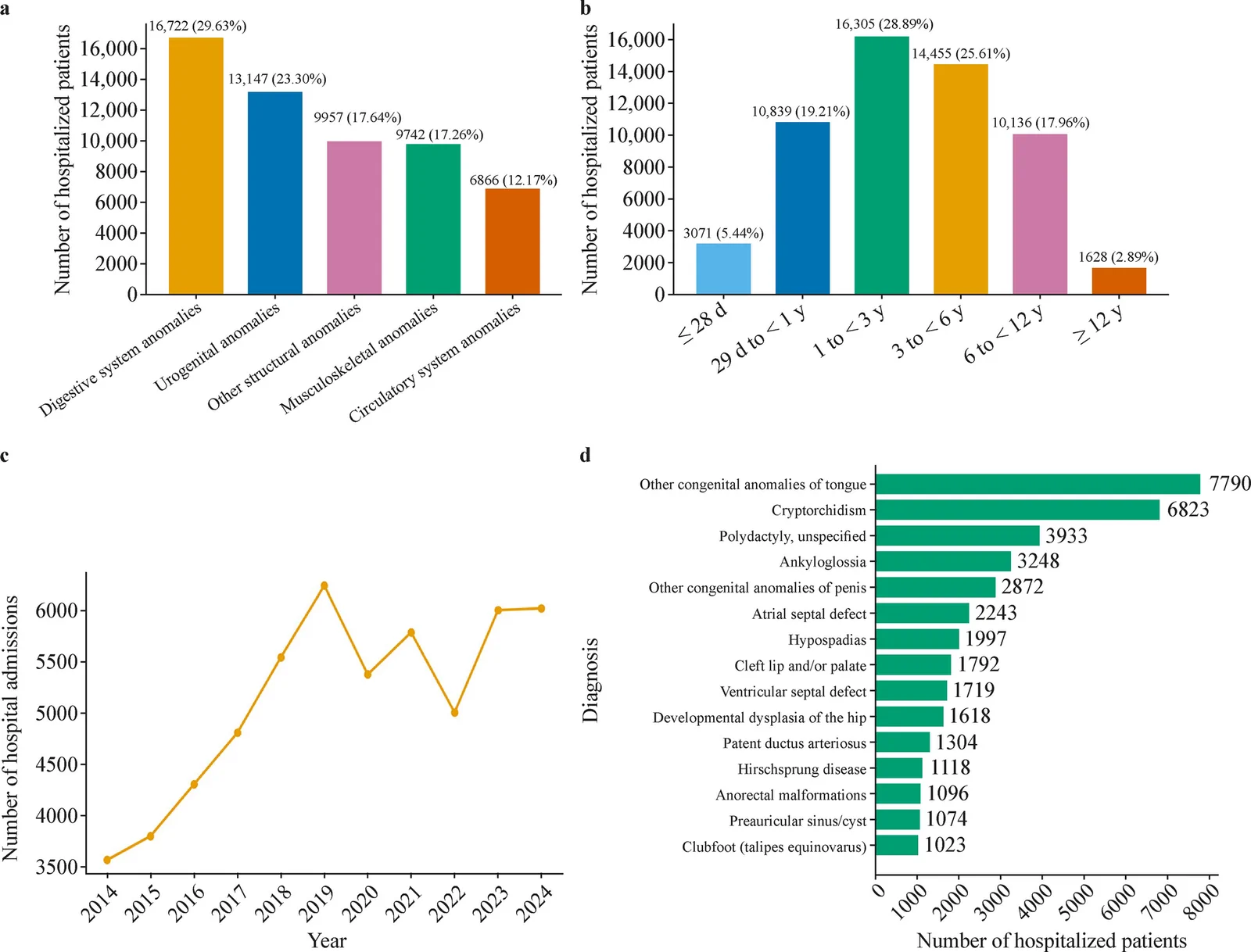
Demographics and disease spectrum of pediatric inpatients with congenital structural anomalies, 2014–2024. **a** Distribution of congenital structural anomalies by major organ system among hospitalized children; **b** age distribution of pediatric inpatients with congenital structural anomalies; **c** annual trend in hospital admissions for congenital structural anomalies; **d** top 15 most frequent diagnoses among pediatric inpatients with congenital structural anomalies

Digestive system anomalies accounted for the largest proportion of patients (16,722, 29.63%), followed by urogenital anomalies (13,147, 23.30%). Other structural anomalies comprised 9957 (17.64%) patients, musculoskeletal anomalies 9742 (17.26%), and circulatory anomalies 6866 (12.17%). The relative contributions of the five major congenital anomaly systems are summarized in Fig. 1a.

Age-stratified analysis showed that patients were predominantly concentrated in early childhood. The 1 to < 3 years age group accounted for the largest proportion (16,305, 28.89%), followed by 3 to < 6 years (14,455, 25.61%) then 29 days to < 1 year (10,839, 19.21%). Children aged 6 to < 12 years accounted for 10,136 (17.96%) patients, whereas neonates ≤ 28 days represented 3071 (5.44%) patients and children aged ≥ 12 years accounted for only 1628 (2.89%) patients. The age distribution of pediatric inpatients with congenital structural anomalies is illustrated in Fig. 1b. Overall, congenital structural anomalies were most frequently diagnosed among infants and preschool-aged children (Table 1).

Analysis of annual admissions revealed a steadily increasing hospitalization burden over the study period, rising from 3568 patients in 2014 to a peak of 6237 in 2019, followed by a transient decline in 2020 (5374) and a subsequent increase to 6017 in 2024. This temporal trend in annual hospitalizations is shown in Fig. 1c.

The most frequent individual diagnoses included other congenital tongue anomalies (7790), cryptorchidism (6823), and polydactyly (3933), followed by ankyloglossia, other congenital penile anomalies, atrial and ventricular septal defects, hypospadias, cleft lip and/or palate, developmental dysplasia of the hip, and patent ductus arteriosus. The distribution of the top 15 diagnoses is presented in Fig. 1d.

### Geographically weighted regression analysis

Among the 56,434 pediatric inpatients, 41,531 with high-frequency congenital anomalies were included in the spatial analysis cohort to ensure model stability. The system-specific sample sizes were 13,252 for digestive anomalies, 11,692 for urogenital anomalies, 7489 for musculoskeletal anomalies, 5265 for circulatory anomalies, and 3833 for other structural anomalies. Because the primary aim of the GWR analysis was to characterize spatial heterogeneity rather than to conduct location-specific hypothesis testing, conventional global *P* values or county-specific confidence intervals were not used as the primary basis for interpretation. Instead, the results were interpreted using comparative model-performance metrics and descriptive summaries of local coefficient distributions.

**Fig. 2:**
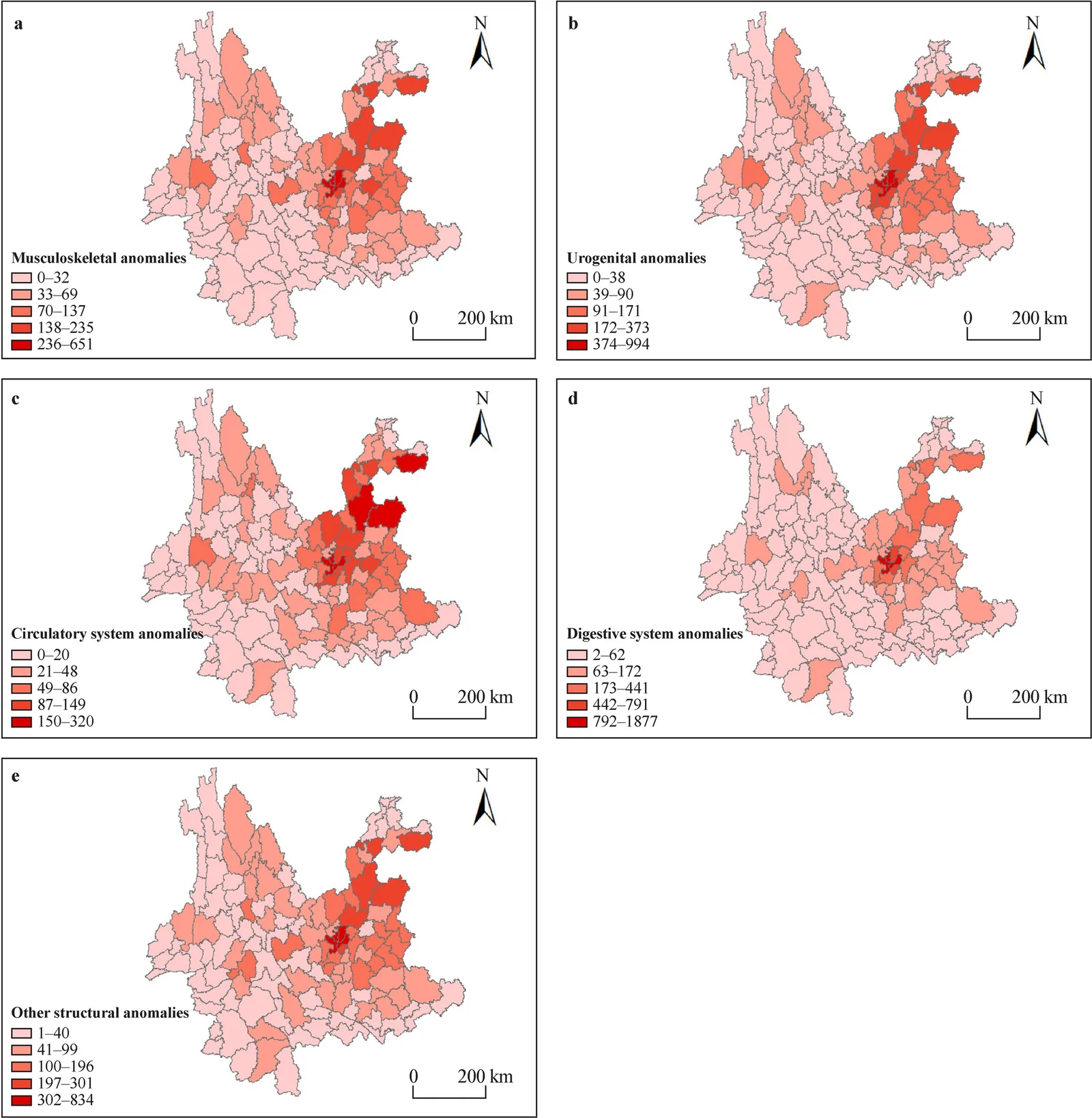
Geographic context and regional distribution of hospital-based institutional burden of congenital structural anomalies in Yunnan Province. **a** Musculoskeletal anomalies; **b** urogenital anomalies; **c** circulatory system anomalies; **d** digestive system anomalies; **e** other structural anomalies. This map was prepared using the standard map downloaded from the Standard Map Service website of the Ministry of Natural Resources of the People's Republic of China [review number: GS(2023)2767]. The boundaries of the base map have not been modified, and a linear scale bar was used in accordance with relevant map-publication requirements

To provide geographic context for subsequent spatial analyses, we summarized the county-level distribution of referral-weighted institutional burden across Yunnan Province. As shown in Fig. 2, institutional case counts were unevenly distributed across counties, with higher case volumes observed in more densely populated and economically developed areas. These descriptive maps contextualize spatial heterogeneity in institutional burden and referral patterns rather than indicating population-based disease risk.

**Fig. 3:**
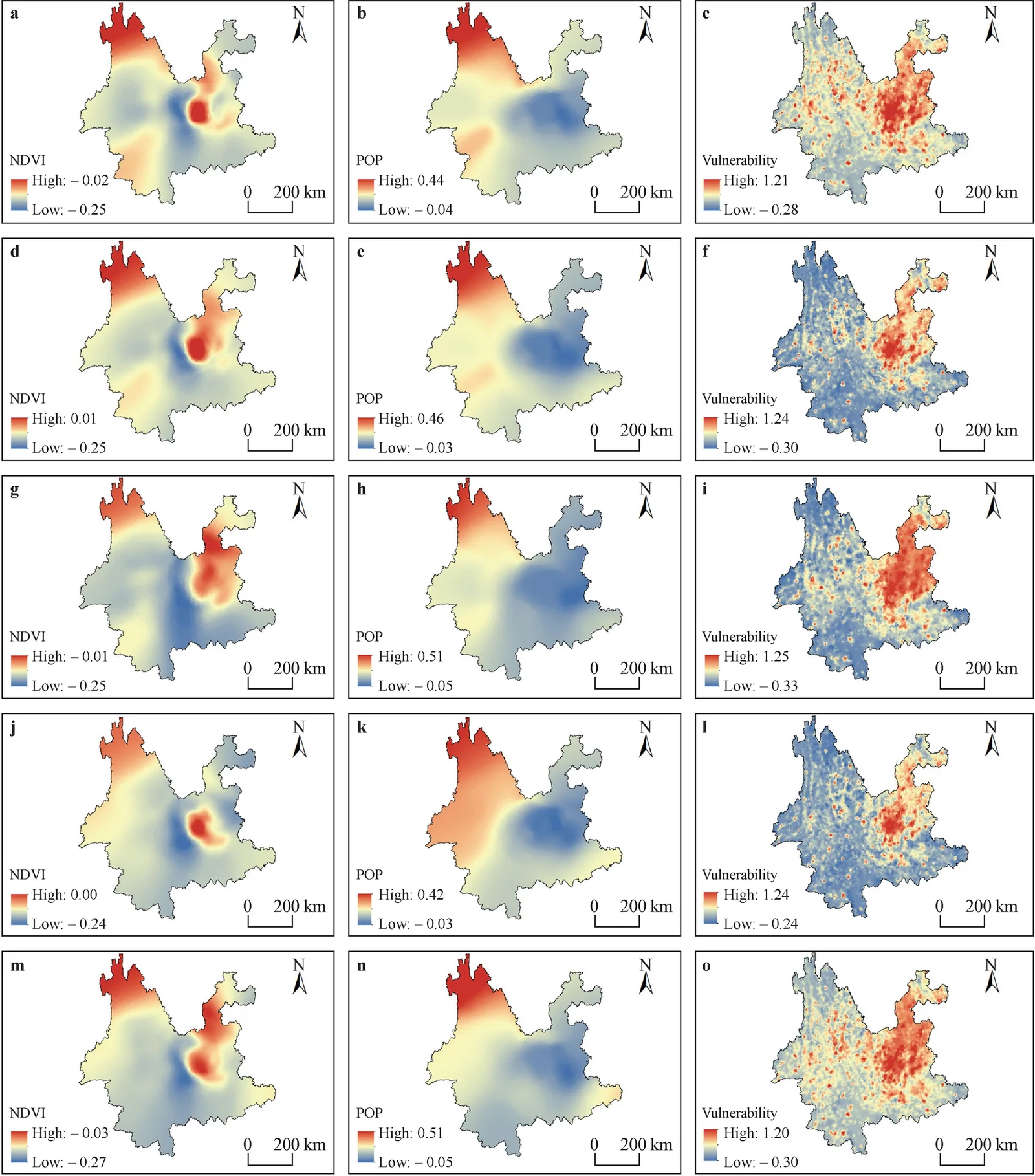
System-specific spatial distribution of environmental and population background variables across five congenital anomaly systems. **a** Digestive system anomalies: spatial distribution of NDVI; **b** digestive system anomalies: spatial distribution of POP; **c** digestive system anomalies: spatial distribution of the composite vulnerability index; **d** urogenital anomalies: spatial distribution of NDVI; **e** urogenital anomalies: spatial distribution of POP; **f** urogenital anomalies: spatial distribution of the composite vulnerability index; **g** musculoskeletal anomalies: spatial distribution of NDVI; **h** musculoskeletal anomalies: spatial distribution of POP; **i** musculoskeletal anomalies: spatial distribution of the composite vulnerability index; **j** circulatory system anomalies: spatial distribution of NDVI; **k** circulatory system anomalies: spatial distribution of POP; **l** circulatory system anomalies: spatial distribution of the composite vulnerability index; **m** other structural anomalies: spatial distribution of NDVI; **n** other structural anomalies: spatial distribution of POP; **o** other structural anomalies: spatial distribution of the composite vulnerability index. *NDVI* normalized difference vegetation index, *POP* population density. This map was prepared using the standard map downloaded from the Standard Map Service website of the Ministry of Natural Resources of the People's Republic of China [review number: GS(2023)2767]. The boundaries of the base map have not been modified, and a linear scale bar was used in accordance with relevant map-publication requirements

Spatial interpolation maps demonstrated marked geographic variability in system-specific background contextual surfaces across Yunnan Province. Figure 3 presents the spatial distribution of NDVI, population density, and a descriptive composite vulnerability index for each anomaly system, providing contextual background for interpreting spatial heterogeneity in subsequent GWR analyses. These maps describe spatial distribution patterns rather than effect estimates, which were subsequently quantified using GWR.

Across all five congenital anomaly systems, GWR models consistently outperformed the corresponding global OLS models, supporting the presence of substantial spatial non-stationarity in area-level associations. As shown in Table 2, GWR models yielded higher coefficients of determination (*R*^2^ and adjusted *R*^2^) and lower AICc and CV values than the global models across all systems. These statistics indicate improved local model fit after allowing coefficients to vary spatially, but they should be interpreted as comparative model-performance metrics for referral-weighted institutional burden rather than evidence of population-level causal effects or county-specific disease risk.

**Table 2:** Comparative performance of global ordinary least squares and geographically weighted regression models across five congenital anomaly systems

Dataset	Preprocessed model observations *(n*)	Bandwidth	ENP [trace (S)]	Global *R*^*2*^	Global AICc	Global CV	GWR *R*^*2*^	GWR adjusted *R*^*2*^	GWR AICc	GWR CV
Musculoskeletal anomalies	14,898	2718.894564	157.576122	0.543975	9953.667151	0.114189	0.639945	0.634829	6727.985659	0.092172
Urogenital anomalies	23,280	4387.224223	163.602549	0.610708	11,855.919750	0.097426	0.700780	0.697960	6035.284697	0.076041
Circulatory system anomalies	26,402	5483.919484	158.433546	0.616137	13,071.602367	0.096059	0.703750	0.701364	6526.068919	0.075162
Digestive system anomalies	10,572	1944.654718	146.433748	0.506937	7895.563120	0.123524	0.623605	0.616582	5314.172123	0.096884
Other structural anomalies	19,824	3600.518578	153.582131	0.560239	12,516.304468	0.110069	0.652671	0.649079	8124.273707	0.088366

*ENP* effective number of parameters, *trace(S)* trace of the geographically weighted regression hat matrix, *AICc* corrected Akaike information criterion, *CV* cross-validation, *GWR* geographically weighted regression. Preprocessed model observations refer to the final observations entered into each system-specific GWR model and should not be interpreted as the number of patients, counties, or unique individuals

Table 3 summarizes the distributions of local GWR coefficients for each environmental predictor across the five congenital anomaly systems. Specifically, the mean coefficient indicates the average direction of the area-level association within each system, whereas the standard deviation, minimum, maximum, and range describe the extent of spatial heterogeneity in the local coefficients. Larger ranges indicate greater non-stationarity, suggesting that the strength and even the direction of the association may vary across locations. These local coefficient summaries are presented for descriptive interpretation and hypothesis generation, rather than for population-level causal inference or formal location-specific effect estimation. Across systems, some predictors showed relatively stable coefficient distributions, whereas others exhibited wider ranges, indicating stronger spatial variability in their associations with referral-weighted institutional burden. This pattern further supports the use of local rather than global models for characterizing geographically heterogeneous environmental associations.

**Table 3:** Summary statistics of local geographically weighted regression coefficients for each environmental predictor by congenital anomaly system

Variables	Musculoskeletal anomalies		Urogenital anomalies		Circulatory system anomalies		Digestive system anomalies		Other structural anomalies	
	Mean (SD)	Range (min–max)	Mean (SD)	Range (min–max)	Mean (SD)	Range (min–max)	Mean (SD)	Range (min–max)	Mean (SD)	Range (min–max)
Intercept	0.845 (0.381)	2.538 (– 0.648 to 1.889)	0.681 (0.468)	3.346 (– 1.074 to 2.272)	0.744 (0.453)	4.959 (– 1.147 to 3.812)	0.855 (0.607)	3.313 (– 1.003 to 2.311)	0.758 (0.529)	2.716 (– 0.634 to 2.081)
SO_2_ (μg/m^3^)	– 0.024 (0.050)	0.332 (– 0.251 to 0.081)	– 0.021 (0.045)	0.342 (– 0.281 to 0.060)	– 0.030 (0.051)	0.375 (– 0.310 to 0.065)	– 0.015 (0.040)	0.224 (– 0.149 to 0.075)	– 0.035 (0.040)	0.265 (– 0.232 to 0.033)
Population density (persons/km^2^)	0.107 (0.102)	0.488 (– 0.044 to 0.444)	0.113 (0.112)	0.496 (– 0.035 to 0.461)	0.117 (0.115)	0.453 (– 0.038 to 0.415)	0.116 (0.122)	0.567 (– 0.058 to 0.509)	0.104 (0.118)	0.575 (– 0.060 to 0.516)
PM_2.5_ (μg/m^3^)	0.005 (0.077)	0.498 (– 0.238 to 0.260)	0.013 (0.068)	0.854 (– 0.162 to 0.692)	– 0.030 (0.068)	0.756 (– 0.393 to 0.363)	0.001 (0.082)	0.531 (– 0.276 to 0.255)	0.017 (0.066)	0.360 (– 0.177 to 0.183)
PM_10_ (μg/m^3^)	– 0.067 (0.057)	0.406 (– 0.350 to 0.056)	– 0.047 (0.049)	0.324 (– 0.260 to 0.064)	– 0.040 (0.042)	0.284 (– 0.255 to 0.029)	– 0.068 (0.051)	0.335 (– 0.260 to 0.075)	– 0.051 (0.040)	0.305 (– 0.282 to 0.023)
O_3_ (μg/m^3^)	– 0.034 (0.045)	0.239 (– 0.170 to 0.069)	– 0.021 (0.045)	0.256 (– 0.126 to 0.130)	– 0.022 (0.046)	0.291 (– 0.165 to 0.125)	– 0.018 (0.052)	0.299 (– 0.130 to 0.169)	– 0.006 (0.051)	0.259 (– 0.116 to 0.143)
NO_2_ (μg/m^3^)	0.067 (0.056)	0.274 (– 0.073 to 0.201)	0.059 (0.051)	0.222 (– 0.033 to 0.189)	0.059 (0.049)	0.239 (– 0.035 to 0.204)	0.060 (0.058)	0.241 (– 0.057 to 0.184)	0.057 (0.051)	0.275 (– 0.059 to – 0.217)
NDVI	– 0.152 (0.059)	0.235 (– 0.257 to – 0.023)	– 0.130 (0.070)	0.266 (– 0.258 to 0.008)	– 0.115 (0.066)	0.246 (– 0.247 to 0.000)	– 0.134 (0.058)	0.244 (– 0.253 to –0.008)	– 0.153 (0.062)	0.243 (– 0.279 to – 0.036)
Land surface temperature (°C)	0.033 (0.062)	0.342 (– 0.082 to 0.259)	0.037 (0.062)	0.451 (– 0.078 to 0.372)	0.029 (0.058)	0.396 (– 0.123 to 0.272)	0.004 (0.064)	0.294 (– 0.150 to 0.143)	0.050 (0.063)	0.361 (– 0.096 to 0.265)
Elevation (m, digital elevation model)	– 0.041 (0.048)	0.356 (– 0.268 to 0.088)	– 0.045 (0.057)	0.375 (– 0.316 to 0.059)	– 0.029 (0.055)	1.201 (– 0.969 to 0.232)	– 0.043 (0.037)	0.224 (– 0.167 to 0.057)	– 0.035 (0.043)	0.272 (– 0.206 to 0.066)
CO_2_ (mg/m^3^)	0.010 (0.097)	0.571 (– 0.265 to 0.306)	0.020 (0.083)	0.470 (– 0.141 to 0.329)	0.013 (0.062)	0.355 (– 0.143 to 0.212)	0.048 (0.109)	0.581 (– 0.136 to 0.445)	0.001 (0.060)	0.431 (– 0.168 to 0.263)
CO (mg/m^3^)	0.048 (0.054)	0.374 (– 0.086 to 0.289)	0.040 (0.048)	0.312 (– 0.099 to 0.213)	0.047 (0.056)	0.524 (– 0.215 to 0.309)	0.052 (0.035)	0.336 (– 0.116 to 0.219)	0.027 (0.039)	0.287 (– 0.098 to 0.189)

*SD* standard deviation*, SO₂* sulfur dioxide, *O₃* ozone, *NO*₂ nitrogen dioxide, *NDVI* normalized difference vegetation index, *CO₂* carbon dioxide, *CO* carbon monoxide

Carbon monoxide exhibited relatively stable positive associations with referral-weighted institutional burden across systems, whereas sulfur dioxide showed pronounced spatial heterogeneity, with local coefficients varying in both magnitude and direction. Vegetation coverage demonstrated a consistently negative association across all systems, while population density showed positive, but spatially variable associations (Table 3).

## Discussion

By integrating a large pediatric inpatient cohort and spatially explicit analytical methods, this study provides a comprehensive depiction of the demographic characteristics and geographically heterogeneous environmental contexts associated with congenital structural anomalies in Yunnan Province [13–15]. Rather than assuming uniform associations across regions, our findings underscore substantial spatial non-stationarity, highlighting the limitations of conventional global models in geographically diverse settings [16, 17].

Although previous studies have proposed possible biological pathways linking prenatal environmental exposures to congenital anomalies, including oxidative stress, placental dysfunction, inflammatory responses, and disruption of embryonic developmental signaling, the present hospital-based ecological analysis was not designed to test these mechanisms directly. Accordingly, the spatial associations observed in this study should be interpreted as contextual, area-level patterns of referral-weighted institutional burden rather than evidence of specific causal environmental effects. Any mechanistic interpretation remains speculative in the context of the current data and requires confirmation in future population-based, multi-center, and mechanism-oriented studies.

The contextual spatial associations observed in hospital-based institutional burden were heterogeneous across anomaly systems. Such heterogeneity likely reflects regional differences in environmental conditions, population distribution, healthcare accessibility, and referral patterns, rather than simple or uniform exposure-outcome relationships [18–20]. These findings are consistent with growing evidence that pediatric health outcomes are shaped by interacting environmental and contextual factors that vary across space [21, 22]. Population-level birth sex ratio data for the hospital catchment region were not available; therefore, direct comparison with the background population was not feasible.

Importantly, this study draws on data from the largest provincial pediatric referral center in Yunnan. As a provincial pediatric referral center serving both urban and remote areas, our institution enables assessment of hospital-based burden and referral-related spatial patterns [23, 24].

These patterns may help generate hypotheses for future surveillance-oriented or multi-center studies, but they should not be interpreted as sufficient evidence for direct policy action without validation in population-based datasets. Uniform approaches may be insufficient in regions characterized by marked environmental and socioeconomic diversity, and spatially explicit evidence may help generate hypotheses for future surveillance-oriented or multi-center studies [25–27].

Several limitations should be acknowledged. First, this was a single-center, inpatient-based study, and the findings primarily reflect referral-related patterns and institutional disease burden rather than population-based epidemiology. Counties with poorer transportation conditions, lower socioeconomic resources, weaker referral linkages, or lower access to tertiary care may be underrepresented in the hospital datasets even if their true burden on congenital anomalies is substantial. Accordingly, the external validity and inferential strength of modeling results derived from a single-hospital dataset are inherently limited, and the observed spatial patterns may partly reflect institutional catchment structure and referral inequality rather than underlying disease occurrence. Second, although the dataset was derived from a hospital information system with routine clinical data recording and standardized diagnostic procedures, potential inaccuracies related to diagnostic coding, referral patterns, and variations in clinical documentation cannot be completely excluded. In addition, classifying patients with multiple anomalies solely by the principal discharge diagnosis may underestimate the total anomaly burden across systems and cannot fully characterize comorbidity patterns. This strategy was adopted to preserve mutually exclusive patient-level categories and reduce information bias arising from incomplete recording or evaluation of secondary anomalies, particularly among critically ill patients who deteriorated or died before complete multisystem assessment. Third, due to the retrospective design and the lack of individual-level maternal residential histories, trimester-specific prenatal exposure assessment was not feasible. Consequently, the observed spatial patterns reflect area-level environmental contexts rather than individual-level causal effects. Fourth, sensitivity analyses using alternative model specifications (for example, count-based or offset-based spatial regression) could not be performed because of the software constraints of the GWR platform used for the original modeling and should be considered in future work using more flexible statistical environments. Fifth, the use of aggregated county-level data may obscure individual-level exposure variability; causal inferences cannot be drawn from the observed associations. Prenatal screening and diagnostic programs, as well as pregnancy termination practices, may influence the representation of severe congenital anomalies in hospital-based datasets, potentially leading to the underrepresentation of certain major malformations. Environmental exposures were estimated using interpolated or aggregated datasets, which may introduce measurement uncertainty. Finally, although ethnicity information was available, detailed socioeconomic, environmental, and genetic covariates stratified by ethnic groups were not available, limiting the ability to assess ethnicity-specific risks or disparities. It should also be emphasized that the spatial tendencies of institutional burden derived from Gaussian GWR fitted values represent relative, model-based patterns rather than individual-level risk or causal effects and are intended to support spatial comparison and hypothesis generation rather than etiological inference.

Future studies incorporating population-based surveillance, birth registries, multi-center clinical data, longitudinal designs, individual-level exposure assessment, and mechanism-oriented investigation will be necessary to validate the robustness of these spatial associations and clarify underlying pathways.

Taken together, this hospital-based study provides an overview of the demographic characteristics of pediatric congenital structural anomalies and highlights spatial heterogeneity in area-level environmental contexts across anomaly systems in Yunnan Province. Local spatial models captured non-stationary patterns more effectively than global models, supporting the value of spatially explicit approaches for characterizing referral-weighted institutional burden. Nevertheless, because the analysis was based on a single-center, referral-weighted institutional dataset rather than population-based surveillance, the findings should be interpreted as descriptive and contextual rather than causal or population-representative. These results provide a hypothesis-generating basis for future pediatric epidemiological research and warrant further validation using population-based or multi-center datasets.

## Supplementary Information

Below is the link to the electronic supplementary material.

Supplementary file1 (DOCX 28 KB)

## Data Availability

The datasets used and/or analyzed in this study are available from the corresponding author on reasonable request. Due to institutional data protection regulations and patient privacy considerations, the raw data are not publicly available.
