# Supplementary material for: Spatial heterogeneity in area-level environmental context of hospital-based congenital structural anomaly burden in Southwest China: a retrospective study from a provincial pediatric referral center
Source: World J Pediatr. 2026 Sep 3;22(8):955–67. doi: 10.1007/s12519-026-01059-w (PMC13615009; doi:10.1007/s12519-026-01059-w)
Supplement: Supplementary file 1 [file 12519_2026_1059_MOESM1_ESM.docx]

# Interpretation of Gaussian geographically weighted regression fitted values

In the Gaussian geographically weighted regression framework, fitted values (*ŷ*) were interpreted as location-specific expected levels of referral-weighted institutional burden after accounting for the contextual covariates retained in each system-specific model. Because the dependent variables were county-linked measures derived from a single provincial referral center, the fitted values were not interpreted as population-based incidence, individual susceptibility, or causal risk. For visualization, fitted values were standardized to facilitate comparison across space within each anomaly system. The resulting mapped surface was therefore treated as a relative spatial tendency of institutional burden index rather than an absolute epidemiological measure.

# Contextual variables and normalized difference vegetation index handling

Environmental covariates were processed as long-term county-level background indicators. Normalized difference vegetation index (NDVI) was derived from the MOD13A3 dataset acquired by the moderate resolution imaging spectroradiometer sensor onboard the Terra satellite and was aggregated to the county level in parallel with the other contextual variables used for spatial modeling. The purpose of the NDVI variable in the present study was to provide a broad measure of regional vegetation coverage and ecological context rather than to estimate individual prenatal exposure windows. All contextual variables were aligned to the county spatial framework before modeling, and multicollinearity was evaluated using variance inflation factors prior to final model selection.

# Descriptive composite vulnerability index

A descriptive composite vulnerability index was generated for visualization only to summarize spatial differences in baseline contextual vulnerability across the study region. Variables selected for this exploratory surface were standardized, directionally harmonized, and overlaid within the geographic information system workflow so that higher values reflected relatively greater contextual vulnerability. Because this index was designed as an exploratory map layer rather than an inferential model output, it was not used for hypothesis testing or causal interpretation.

# Interpretive caution

Both the standardized fitted-value maps and the descriptive composite vulnerability index should be interpreted as contextual and hypothesis-generating visual summaries. These spatial displays are influenced by referral pathways, healthcare accessibility, and institutional catchment structure in addition to background environmental context. Accordingly, they should not be used as substitutes for population-based surveillance or for county-specific causal inference.
